# An overview of patient involvement in healthcare decision-making: a situational analysis of the Malaysian context

**DOI:** 10.1186/1472-6963-13-408

**Published:** 2013-10-11

**Authors:** Chirk-Jenn Ng, Ping-Yein Lee, Yew-Kong Lee, Boon-How Chew, Julia P Engkasan, Zarina-Ismail Irmi, Nik-Sherina Hanafi, Seng-Fah Tong

**Affiliations:** 1 University Malaya Primary Care Research Group, Department of Primary Care Medicine, Faculty of Medicine, University of Malaya, Kuala Lumpur, Malaysia; 2 Department of Family Medicine, Faculty of Medicine and Health Science, Universiti Putra Malaysia, Serdang, Malaysia; 3 Department of Rehabilitation Medicine, Faculty of Medicine, University of Malaya, Kuala Lumpur, Malaysia; 4 Department of Family Medicine, Medical Faculty, Universiti Kebangsaan Malaysia, Kuala Lumpur, Malaysia

## Abstract

**Background:**

Involving patients in decision-making is an important part of patient-centred care. Research has found a discrepancy between patients’ desire to be involved and their actual involvement in healthcare decision-making. In Asia, there is a dearth of research in decision-making. Using Malaysia as an exemplar, this study aims to review the current research evidence, practices, policies, and laws with respect to patient engagement in shared decision-making (SDM) in Asia.

**Methods:**

In this study, we conducted a comprehensive literature review to collect information on healthcare decision-making in Malaysia. We also consulted medical education researchers, key opinion leaders, governmental organisations, and patient support groups to assess the extent to which patient involvement was incorporated into the medical curriculum, healthcare policies, and legislation.

**Results:**

There are very few studies on patient involvement in decision-making in Malaysia. Existing studies showed that doctors were aware of informed consent, but few practised SDM. There was limited teaching of SDM in undergraduate and postgraduate curricula and a lack of accurate and accessible health information for patients. In addition, peer support groups and 'expert patient’ programmes were also lacking. Professional medical bodies endorsed patient involvement in decision-making, but there was no definitive implementation plan.

**Conclusion:**

In summary, there appears to be little training or research on SDM in Malaysia. More research needs to be done in this area, including baseline information on the preferred and actual decision-making roles. The authors have provided a set of recommendations on how SDM can be effectively implemented in Malaysia.

## Background

Involving patients in decision-making is a good clinical practice and, in some countries, it is imperative for routine patient care [1-4]. This forms part of patient-centred care and is increasingly considered to be the gold standard of medical care by the public, clinicians, and policy makers [4]. There is growing evidence, suggesting that involving patients in decision-making helps improve their knowledge and healthcare experience and reduce health service utilisation and cost [5]. The evidence also suggests that patients may modify their health behaviour and status after being involved in decision-making [5].

Focus on decision-making has led to the development of the shared decision-making (SDM) model, in which patients and doctors share information and values, and patients play an active role in making healthcare decisions [6,7]. However, the concept of SDM is interpreted differently in various social and cultural contexts. For example, a recent review found wide SDM practice and policy variations across 13 countries in the Middle East, Europe, and North and South America [8]. Thus, implementing SDM remains challenging, even in countries where SDM is officially endorsed by government, such as the United Kingdom and the United States of America [1,9,10]. Practising SDM is even more challenging in countries that have scarce healthcare resources and an overburdened healthcare system, which are common in Asia. Despite these challenges, there is a growing interest in SDM globally in terms of “scope (as a component of patient-centred care) and spread (as a component of healthcare everywhere for everyone)” [11].

In Asia, there is limited knowledge of how the SDM concept has been, or could be, integrated into existing practice. At a micro level, little is known about the decision-making role preference of patients and physicians. At a macro level, it is uncertain whether the Western model of SDM is transferable to societies where healthcare decisions of individuals are strongly influenced by their families and communities [12]. Asia is not a homogenous continent; for instance, healthcare decisions of Chinese, Japanese, and Vietnamese people are influenced by diverse concepts of harmony and filial piety, which originate from different religious or moral codes [13]. In 2005, Charles argued that SDM should not be practised without considering the cultural context, of a clinical consultation [14]. Studies with ethnic minorities in the West have identified the challenges in practising SDM, particularly in communities where the concept of SDM is foreign or non-existent [15-17].

There is one assumption that people in the East prefer a more clinician-centred healthcare system, though there is a lack of evidence. A recent survey in Japan shows that patients want to be more involved in healthcare decision-making [18]. Although there are still significant differences between Western and Asian cultures, globalisation and advancement of telecommunication have blurred distinctions significantly over the past two decades. Moreover, the overall improvement in literacy rates and health awareness mean that public health expectations are rising in Asia [19,20].

Therefore, it is prudent and timely to review the current research evidence, practices, policies, and laws with respect to SDM in Asia. This article uses Malaysia, a multi-cultural Asian society, to exemplify the existing and emerging issues of SDM in the areas of education, clinical practices, and healthcare policies in Asia.

Malaysia has a population of 28.3 million and comprises three main ethnic groups: Malays (67.4%), Chinese (24.6%), Indians (7.3%), and others (0.7%) [21]. Malaysia is classified by the United Nations as an upper-middle income nation and has a dual sector (public and private) healthcare system. People pay a nominal fee for public health services, which are often overburdened and have long waiting times. On the other hand, the private health sector charges a fee for services and people can choose the hospital, clinic, and healthcare professionals. A multi-cultural society and a dual-sector health system in Malaysia provide an opportunity to study Asian patients’ involvement in decision-making, using the SDM concept to analyse the structures that form decision-making practice and policy.

## Methods

The authors met in July 2011 and came to consensus on five key areas, which reflect the condition of patient involvement in the Malaysian healthcare system. The five categories reviewed were (1) clinical training and education, (2) research, (3) patient information and support, (4) laws and regulations, and (5) health policies.

### Study design

This study comprised of an environmental scan followed by group consensus methods. In the environmental scan, four sources were used to determine the status of patient involvement in Malaysia. The research group then met to discuss the findings and formulate strategies for increasing SDM in Malaysia.

### Sources of data

As the study covers a wide range of objectives, a range of data sources were used to determine the status of patient involvement in decision making in Malaysia. These sources include (1) academicians from main public universities in Malaysia; (2) databases searched for literature review; (3) patient support groups and review of governmental and non-governmental web sites on health information for patients; and (4) Malaysian laws and health policies.

### Identification of eligible material

The study aimed to include any data or information on the following key areas: SDM training and education; research and clinical practice of SDM; patient information and support; legislations and policies on or related to SDM.

### Data extraction

The following methods were used to collect data from the four sources: (1) an online survey with academicians from main public universities in Malaysia; (2) a comprehensive literature review of patient involvement in decision making; (3) an online survey of patient support groups and review of governmental and non-governmental web sites on health information for patients; and (4) a document review of Malaysian laws and health policies.

#### The online survey on clinical training and education in SDM

We wrote emails to 15 academicians in eight most established public (n = 6) and private medical schools (n = 2) to seek information on training and education. The participants were selected based on their active involvement in undergraduate and postgraduate teaching in their institutions. The participants were asked to provide information on whether the patient involvement and SDM were included in the medical curriculum and, if so, to what extent they were being implemented in practice. Descriptive data using simple frequency count was used to capture the extent to which SDM was incorporated into the medical curriculum.

#### A comprehensive literature review on research and practice of SDM

We searched PubMed, CINAHL, and MyJurnal (a database of Malaysian publications) to identify SDM-related publications up to March 2013. Our search strategies were as follows:

• PubMed: “(patient-centred care OR decision-making OR shared decision-making OR patient participation) AND (Malaysia)” and “patient preference [MeSH] AND Malaysia” (n = 162)

• CINAHL: “(patient-centred care OR shared decision-making OR decision-making OR patient participation) AND (Malaysia)” (n = 105)

• MyJurnal “patient” (n = 995).

Both qualitative and quantitative studies were included in the review. Published articles which met the following criteria were considered for inclusion: qualitative or quantitative studies which collected original data; performed in a healthcare setting; published in English; and those that reported how patients were involved in medical decision-making. Studies that reported patient beliefs and levels of knowledge were excluded. Only studies published as full text articles were included in the review. Review articles were also excluded as they did not report any original data. Duplicates and non-relevant references were removed. One of the researchers identified the relevant articles which were reviewed, extracted and synthesised.

#### Online survey of patient support group and review of official websites for patient health information

We sought information regarding patient involvement in decision-making from four established patient support groups for: diabetes, systemic lupus erythematosus, drug users, and HIV infection. These groups were chosen as they were the few most established support groups in Malaysia. We gathered information from these groups by conducting an informal email survey, enquiring about existing programmes for patient decision support from both healthcare professionals and peers. For patient information and support, we systematically searched the official web sites of the Ministry of Health [22], main public and private medical centres, and healthcare-related non-government organisations. The amount and quality of patient information were appraised according to: the scope of health topics covered by the website; language available (English, Malay, Mandarin, Tamil); user-friendliness (readability); and patient involvement in the development of the health depository.

#### Document review of the laws and policies on SDM

For standards and policies, we reviewed legislations and policies enacted by the Malaysian Medical Council, which is the official body for medical policy, legislation, and regulation in Malaysia. The relevant sections which described patient involvement were extracted and described in the results.

### Data analysis

Simple descriptive analysis was use to describe the data collected from the various sources.

### Group consensus methods

The group corresponded via email to discuss and compile the findings of the environmental scan. Based on the findings, a strategy to increase awareness and implement SDM in Malaysia was formulated (Table 1).

**Table 1:** Proposed strategy to increase awareness and implement SDM in Malaysia

	**Proposed strategy**	**Description**
**1**	**Education**	● Incorporating teaching of SDM into undergraduate curriculum
		○ General communication and consultation skills
		○ Risk communication
		○ Evidence-based medicine
		● Incorporating a more structured SDM teaching into postgraduate curriculum
		○ Communication and consultation skills
		○ Emphasis on specific areas requiring informed consent such as surgeries, chemotherapy, screening
		○ Assessment of trainees competency in SDM
		● Incorporating SDM training into continuing professional development, including workshops on SDM and how to use patient decision aids
**2**	**Clinical practice**	● Incorporating SDM in clinical practice guidelines
		● Advocate the use of patient decision aids or other decision support tools in patient care
		● Patient involvement in decision making as a quality indicator
		● Payment/reimbursement for practices which implement SDM or use decision aids
**3**	**Research**	● Baseline research on patient involvement in decision making at the national level
		● Exploratory studies on the factors influencing decision making in a multi-cultural and multi-lingual context
		● Developing and evaluating decision support interventions to help patients make informed decisions
		● Develop and evaluate interventions to incorporate SDM in routine care
**4**	**Policy and law**	● Malaysian Medical Council should consider developing a national healthcare policy on SDM
		● The Ministry of Health should improve on the existing patient health information system to make the content more accurate, user-friendly and accessible to the public
		● Public health campaigns should target at empowering people to be more involved in their health care and making decisions about their health care

## Results

### SDM training and education

Teaching SDM was not explicitly stated as an objective in most undergraduate and postgraduate curricula in Malaysia. Only one medical school mentioned SDM in their primary care curriculum. However, how SDM is being taught was not clearly defined and evaluated. The process of SDM, such as sharing information, offering treatment choices, exploring patient preferences, involvement of family in decision-making, and sharing the decision, was taught as part of other components of the training programme. For example, risk communication is taught under evidence-based medicine; information sharing and exploring patients’ ideas, concerns, and expectations form part of the communication and consultation skill training; and respecting patients’ autonomy and providing them adequate and accurate information to make an informed decision are taught in medical ethics and informed consent. Feedback from the respective postgraduate coordinators of the discipline of Family Medicine highlighted a lack of structured SDM teaching. Most commented that SDM is being taught as part of the communication and consultation skill training. Overall, structured teaching of SDM in Malaysia is non-existent and, at best, fragmented.

### Research and clinical practice of SDM

We identified 1262 articles, of which 20 focused on SDM or patient involvement in decision-making [23-42]. Studies focused on the areas of informed consent, patient autonomy, decisional role, and the information needs of patients with diabetes, children, the elderly and patients living with cancer. Research on SDM in Malaysia remains scarce. Data suggest that there is a lack of information available for patients to make an informed choice and patients and their parents are not actively involved in decision-making. Overall, despite patient’s desire for quality information [37,38,41] patients were not given enough information to make an informed choice [28,30,43]. Although healthcare professionals, mainly doctors, were aware of the importance of taking informed consent, some would choose not to practise it if diagnosis was unfavourable or if truth was deemed harmful [23,27].

Levels of patient-centredness varied amongst medical specialities [42]. Among the Malaysian paediatric populations, the practice of SDM was even less. Only 20% of the decisions on resuscitation of pre-term babies were made by parents, whereas 72% and 8% of the decisions were made by the physician and the ethics committee, respectively [24]. Similarly, Mazlina and Julia found that most (58%) of the rehabilitation physicians practise medical paternalism and override a patient’s earlier directive to withdraw life-sustaining treatment [31]. Efforts that encourage patient participation include engaging healthcare practitioners in self-management programmes [40] and training on patient-centredness [34].

### Patient information and support

Patient education is an important step towards empowering patient involvement in decision-making. Accessibility to accurate, relevant, and readable health information increases health literacy and engages patients in the discussion of choosing the best option for their health. Low health literacy rate may be an important contributing factor to the lack of patient involvement in decision-making in Malaysia [29].

The Ministry of Health is the main provider of patient health education resources in Malaysia. It recognises the importance of disseminating “accurate, appropriate, and relevant information in a timely, equitable, and innovative manner” and “empowerment of individuals and communities to enable them to take action on the determinants of health” [44]. The Ministry has established a health education Web site for the public [22]. However, the development process of these educational materials is not clear and only limited health topics are covered (obesity, physical activity, smoking, diabetes, heart disease, and mental health). The Web site provides an interactive risk calculator and helps users discuss their results further with doctors. However, SDM is mentioned neither implicitly nor explicitly. Moreover, the usability, the usefulness, and the comprehensiveness of the health information of this Web site have not been evaluated. We are also not sure of the extent to which consumers were involved in the development process. Currently, the Web site is available only in two languages, that is, English and Malay; however, it is not available in Chinese and Tamil, which are spoken by up to one-third of the population. Besides the Ministry of Health Web site, other patient information resources are scattered and are mainly produced by private medical centres or voluntary and patient support groups.

Currently, there are no structured peer support or 'expert patient’ programmes in Malaysia. Most programmes involve patients as volunteers or counsellors, providing emotional support rather than peer education. However, some patient support groups and organisations, such as the National Diabetes Institute, Malaysia, are pursuing links with international peer support organisations, such as Peers for Progress [45], to empower patients to care for themselves and their peers. The recent clinical practice guideline for breast cancer involved breast cancer survivors in the development process [46].

### Legislations and policies on SDM

The Malaysian legislation follows the British common law and the main conflict in SDM involves the concept of consent to care [47]. According to the law, patients must have sufficient information regarding the specific condition he or she is suffering from and the nature and purpose of care being recommended before giving the consent. It is the patient’s right to know and the doctor’s responsibility to warn the patient about the risks (that would make a significant difference in the patient’s life if they materialise) when undergoing or refusing any proposed care [48]. In Malaysia, informed consent is often not practised [23] because of a lack of doctor–patient communication [47].

The Malaysian Medical Council published a guideline on duties of a doctor in 2001, which outlined the moral and professional obligations expected of a medical practitioner in Malaysia [49]. The guideline states that the relationship between a doctor and a patient should be “collaborative” and they should be in a “partnership”. It reaffirms the importance of the doctor–patient relationship, which “paves the way for frank discussion in which a patient’s needs and preferences and a doctor’s clinical expertise are shared to select the best treatment option”. The doctor is also required to “give relevant options when discussing treatment, and the limitations and possible complications”. These recommendations concur with the principle of SDM, where information is exchanged and decisions are made based on a shared understanding and agreement between the two parties.

## Discussion

This study identified the gap in the research, practice, policies and laws related to SDM in Malaysia. The findings from the limited research studies on SDM revealed a low health literacy rate among patients, which may be attributed to, or compounded by, inadequate health information. Medical practitioners still play a paternalistic role in making healthcare decisions for patients and they do not involve patients in decision-making. It is also noted that these studies involved patients of extreme ages (children and elderly) as well as those with life-limiting illnesses. There is a lack of data on how adult patients are involved in making diagnostic or treatment decisions in various clinical settings. Most studies looked at SDM from the perspective of healthcare professionals. None of the studies looked at how patients prefer to be involved in decision-making. In a cross-sectional study involving patients from 11 European countries, there was a significant difference between what decisional roles patients wanted to have and how they were involved in decision-making in the actual clinical encounter [5]. Therefore, future studies should look at patients’ preferred roles and their healthcare experiences in decision-making. This will provide a definitive answer to the question of how Asian patients prefer to be involved in healthcare decision-making.

There was an increasing interest in the development, evaluation, and implementation of SDM in clinical practice and undergraduate and postgraduate curricula. However, efforts were fragmented and not ideal. Teaching and learning of SDM are essential in cultivating a patient-centred approach to healthcare and should be an integral part of the medical curriculum across all disciplines.

In addition, the practice of SDM is complicated by the cultural and language diversity in Malaysia. Doctors not only have to understand patients’ personal and cultural values, but also have to communicate in a language that may not be their mother tongue. Risk communication, negotiation, and achieving agreement require high-level communication skills and demand high language proficiency. Moreover, the public–private dual system results in practice variations. Both factors make the implementation of SDM a challenging task. Future research should focus on effective ways to improve cross-cultural communication and consultations across the two sectors. Interventions to improve SDM, such as patient decision aids, may play a role in reducing practice variations.

Health literacy remains low in Malaysia, which could contribute to the lack of patient involvement in decision-making [29]. Studies have found that improving health literacy empowers patients to play a more active role in managing their health [20,50]. Patients who know about their health problems and respective treatments are more likely to be involved in making healthcare decisions [51-53]. The quality of local health information is generally poor and this is compounded by the lack of translation into common languages. This poses a significant barrier to increasing health awareness and improving health literacy. Government organisations, non-government organisations such as patient and professional bodies, and academic institutions should work together to improve the quality of, and access to, patient information.

Although SDM practice is endorsed by the Malaysian Medical Council, its implementation remains challenging. This requires the council to work closely with the stakeholders, namely the Ministry of Health, professional bodies, patient support agencies and researchers, to develop a strategy to increase the awareness and the implementation of SDM in Malaysia. SDM should be incorporated in the policies to drive changes within the healthcare system. An example is the Washington State Legislation that approved the use of decision aids and SDM when provided with treatment choices [54]. Currently, there is no health policy in Malaysia that specifically addresses the issues related to SDM. National clinical practice guidelines suggest only the involvement of patients in making medically informed decisions. The council should consider the experiences of countries that have existing healthcare policies on SDM, such as the United States of America, Germany, the United Kingdom, and the Netherlands, as well as that of established SDM institutions and bodies, such as the International Patient Decision Aid Standards Collaboration [55], the Health Foundation [56], and the Foundation for Informed Medical Decision Making [57].

There are limitations in this study. Firstly, limited data sources have been used in this study, which comprise mostly secondary data such as literature and Web pages. We did not consider grey literature such as reports and dissertations for this study. Secondly, our results on SDM training and education are based on a convenience sample, which comprised lecturers in the primary care medicine departments only and not in other disciplines.

## Conclusion

In summary, there appears to be little training or research on SDM in Malaysia. More research needs to be done in this area, including baseline information on the preferred and actual decision-making roles. The authors have provided a set of recommendations on how SDM can be effectively implemented in Malaysia.

## Competing interests

The authors declare that they have no competing interests.

## Authors’ contributions

NCJ led the study, compiled and wrote the final manuscript. JPE, PYL, BHC, and SFT collected data on the medical syllabus and training. PYL reviewed official Web sites of government, non-governmental organisations and private healthcare facilities for public information on SDM. YKL conducted the literature review on SDM and patient involvement in Malaysia. ZII reviewed legislation related to SDM and patient involvement. NSH reviewed policies related to SDM and patient involvement. BHC conducted e-mail correspondence with national patient bodies to enquire about patient involvement and support. All authors read and approved the final manuscript.

## Pre-publication history

The pre-publication history for this paper can be accessed here:

http://www.biomedcentral.com/1472-6963/13/408/prepub
